# Cardiovascular Comorbidities in Atopic Dermatitis: A Case Report on the Impact of Dupilumab on Skin and Heart Function

**DOI:** 10.7759/cureus.89586

**Published:** 2025-08-07

**Authors:** Pawel Gluszak, Sandra Wazniewicz, Lidia Chmielewska-Michalak, Pervana Kandola, Dorota Jenerowicz

**Affiliations:** 1 Department of Dermatology, Poznan University of Medical Sciences, Poznan, POL; 2 Department of Cardiology, Poznan University of Medical Sciences, Poznan, POL

**Keywords:** acute eosinophilic myocarditis, atopic dermatitis (ad), cardiovascular comorbidities in atopic dermatitis, dupilumab, medical comorbidities, new onset cardiomyopathy

## Abstract

Atopic dermatitis (AD) is a chronic inflammatory skin condition often complicated by cardiovascular comorbidities, impacting treatment options and outcomes. In this paper, we present a 41-year-old patient with severe AD, asthma, and chronic heart failure, who responded well to dupilumab, showing significant improvements in skin severity scores and heart function. This case underscores the effectiveness of dupilumab in managing AD alongside complex comorbidities.

## Introduction

Atopic dermatitis (AD), known as atopic eczema, is a chronic, recurrent inflammatory dermatosis most commonly appearing in childhood. It is characterized by the occurrence of erythematous and scaly lesions with a predilection for flexural surfaces, accompanied by severe pruritis [1]. It is the third most common dermatosis, estimated to affect 10-20% of the developed world's population [1]. AD occurs in about 10-30% of children and 2-10% of adult patients and has a significant impact on the quality of life [2]. Patients with AD often develop other atopic comorbidities such as asthma, hay fever, food allergy, and eosinophilic esophagitis (EOE). Among nonatopic comorbidities, they have a higher risk of anxiety, depression, suicidality, infections, and cardiovascular disease. All comorbidities should be considered before making a therapeutic decision [3]. In addition, it has been shown that patients with AD have an increased risk of heart failure (HF). This is linked to the aspect of AD and HF, which involves chronic systemic inflammation, however, the exact mechanism is not fully elucidated. Thus, researchers suggest the importance of early treatment of AD in order to prevent the development of HF [4]. However, the presence of multiple cardiovascular comorbidities may complicate the selection of an appropriate therapeutic regimen. For patients with moderate to severe atopic dermatitis (AD), the primary systemic treatment is immunosuppression with medications such as cyclosporine, a calcineurin inhibitor. However, cyclosporine is contraindicated in patients with cardiovascular comorbidities due to its well-documented adverse effects, including an increased risk of hypertension and nephrotoxicity. In such cases, alternative treatments like dupilumab may be appropriate [5]. Dupilumab is a fully human monoclonal antibody that has been shown to reduce and downregulate pro-inflammatory cytokines such as interleukin-4 (IL-4) and interleukin-13 (IL-13), which are considered key drivers of AD pathophysiology. We present a case report of a patient with atopic dermatitis coexisting with several comorbidities who was successfully treated with dupilumab.

## Case presentation

**Figure 1:**
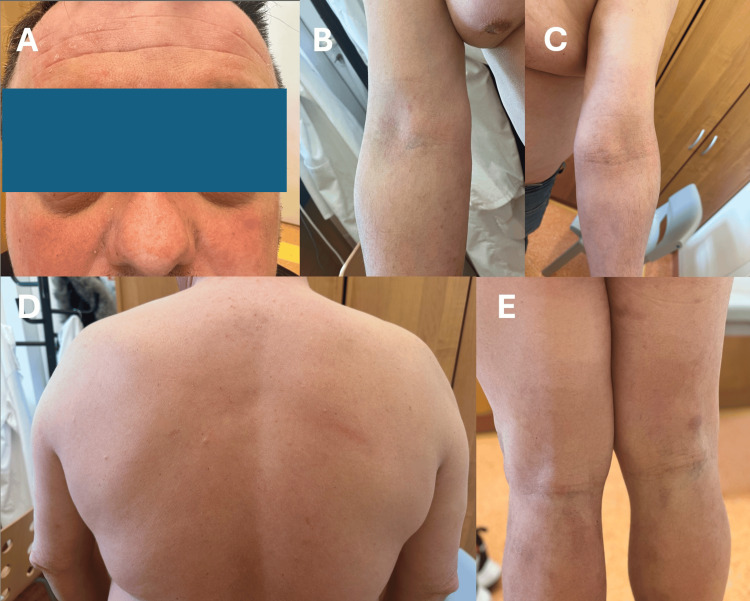
Clinical presentation of a patient with atopic dermatitis Patient demonstrating typical erythematous, inflammatory skin lesions: periorbital erythema and edema (A); erythematous patches in the antecubital fossae (B, C); mild erythema and lichenification on the dorsal trunk (D); erythema and lichenification in the popliteal fossae (E).

A 41-year-old male presented to the dermatology clinic with severe AD since early childhood. He had a history of very severe disease with erythrodermic exacerbations. Previous treatment consisted of topical corticosteroids, emollient therapy, and tacrolimus in 0.1% ointment, and no previous use of systemic immunomodulatory treatment. Medical history included bronchial asthma and cardiovascular comorbidities; the patient was hospitalized in 2003 due to myocarditis, which resulted in the subsequent development of restrictive cardiomyopathy and heart failure. He had no family history of atopic dermatitis or heart failure of any cause. The patient had been hospitalized for an exacerbation of chronic heart failure with an ejection fraction (EF) of 21% in April 2022, with subsequent improvement in EF to 38% in May. Dermatological examination revealed generalized erythroderma, xerosis, and lichenification. The clinical presentation is shown in Figure 1.

**Figure 2:**
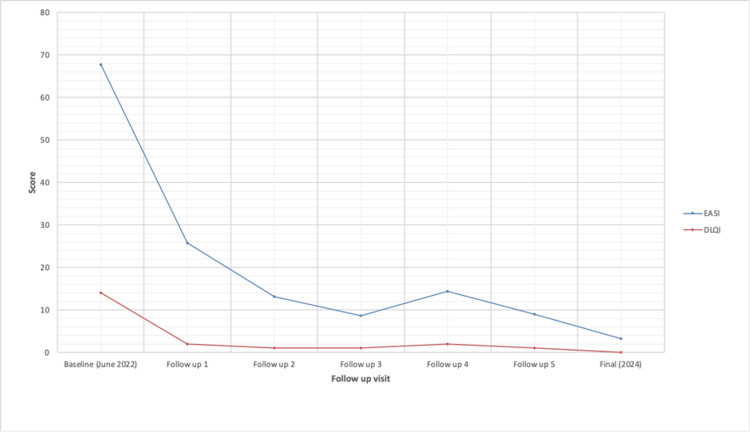
Eczema Area and Severity Index (EASI) and Dermatology Life Quality Index (DLQI) progression after dupilumab initiation. The figure shows the improvement in both EASI and DLQI over time since the initiation of dupilumab at the baseline point in June 2022.

**Figure 3:**
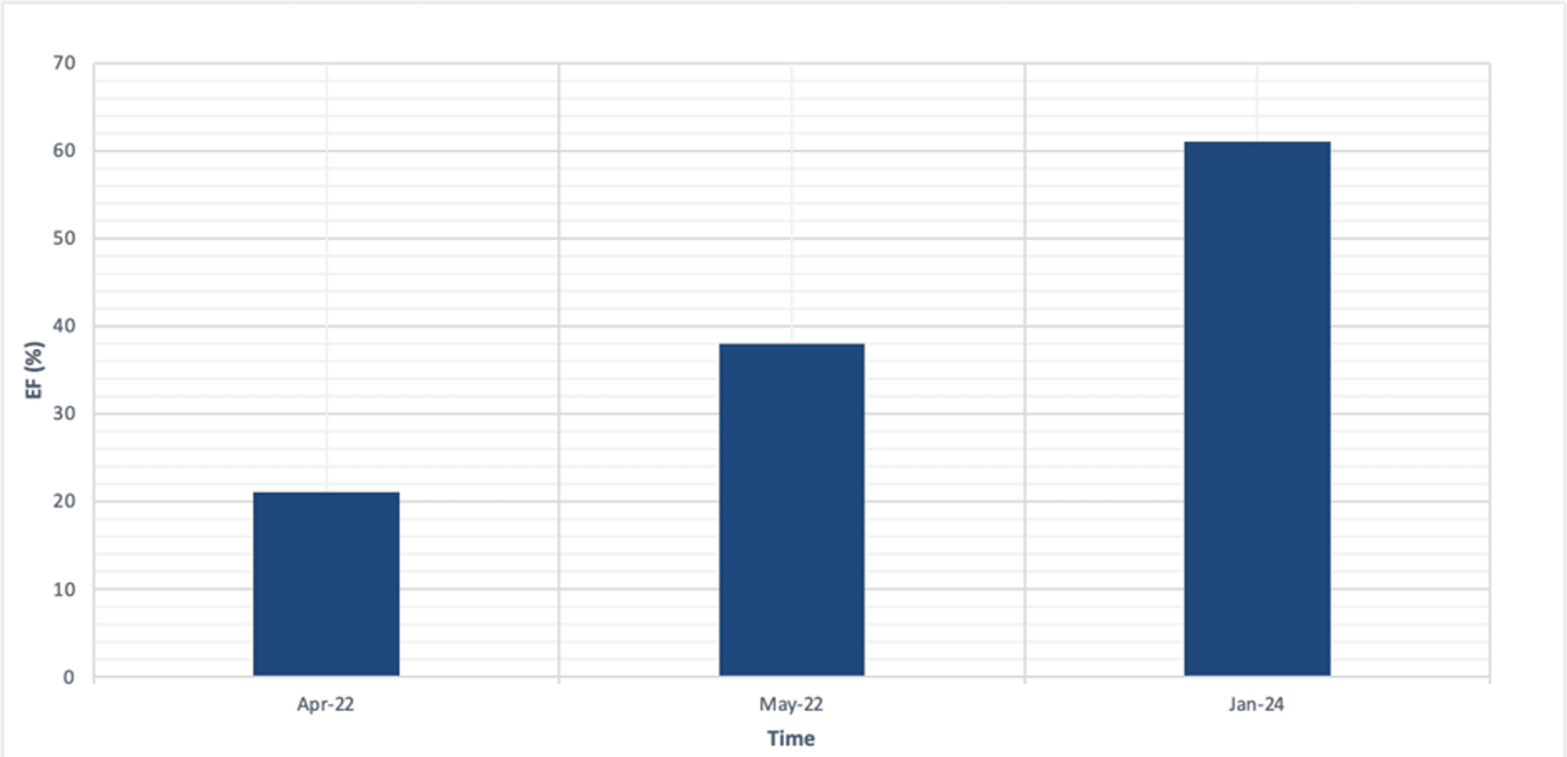
Ejection fraction % (EF%) progression since the initiation of dupilumab. The EF% increased from 21% in April 2022 to 61% in 2024 after the initiation of dupilumab.

Eczema Area and Severity Index (EASI) was 67,8, and Dermatology Life Quality Index (DLQI) was 14. The patient was treated with: torasemide, eplerenone, sacubitril/valsartan, metoprolol, acetylsalicylic acid, and trimetazidine. Given the patient’s diagnosis of heart failure and the associated risk of hyperkalemia when co-administered with multiple heart failure and hypotensive medications, cyclosporine was excluded from the treatment regimen. After a consultation with a cardiologist, due to the lack of contraindications, the patient was qualified for treatment with dupilumab in an initial dose of 600 mg followed by 300 mg every two weeks. The patient underwent a follow-up evaluation five times, achieving a reduction in EASI at consecutive control points: 62%, 80.68%, 87.32%, 78.76%, 86.73%, and DLQI in the range of 1 to 2. At the follow-up visit in 2024, the patient reported significant asthma and cardiac improvement with an EF of 61%, DLQI was 0, and EASI was 3.2. There were no serious side effects except meibomianitis, which subsided after antibiotic therapy. The administration of dupilumab is continued every two weeks. The patient currently remains under the long-term care of a dermatology and cardiology clinic, and the patient reported being extremely satisfied with the treatment and their improved quality of life. Improvement in EASI, DLQI, and EF throughout the course of treatment is documented in Figures 2-3. 

## Discussion

Allergic diseases, such as AD and asthma, leading to a systemic inflammatory response, are risk factors for HF [6]. This association was shown previously by Ascott et al., who identified a dose-response relationship between the severity of AD and cardiovascular disease, especially heart failure [7]. This is further supported by a population-based matched cohort study in 2018, which sub-categorizes the risk of cardiovascular disease. Severe AD patients faced a 20% higher risk of stroke and a 40-50% increased risk of conditions like atrial fibrillation, unstable angina, myocardial infarction, and cardiovascular death. Most significantly, AD patients had a 70% increased risk of heart failure [8]. This association indicates that the management of AD can be affected by cardiovascular comorbidities that can impact treatment options and their efficacy.

AD is due to both genetic and environmental factors. The pathophysiology involves the penetration of allergens through the damaged epidermal layer, resulting in the production of proinflammatory cytokines and a T cell-mediated immune response. IL-4 and IL-13 are mediators that play a key role in the hallmark symptoms of AD as they directly stimulate sensory neurons involved in the neurogenic itch [9,10]. Although the mechanism behind the association between AD and cardiovascular diseases remains unclear, it is postulated that inflammation may play a role specifically as a consequence of the disrupted skin barrier in AD, which is what induces inflammatory responses and oxidative stress via the increased production of reactive oxygen species [4]. Inflammation and oxidative stress play a fundamental role in inducing cellular damage, which can lead to heart failure. However, this correlation remains inconclusive [11]. Another possible cause of heart failure after myocarditis in patients with AD could be Loeffler endocarditis, which is a rare, restrictive cardiomyopathy associated with hypereosinophilia. Among non-cancer-related causes lie allergic diseases, such as AD or asthma, that may result in eosinophil-mediated cell injury [12]. It is usually seen in patients with eosinophilic granulomatosis with polyangiitis (Churg-Strauss syndrome). Animal studies indicate that myocarditis can progress to heart failure and dilated inflammatory cardiomyopathy, not via interleukin-5 (IL-5) - a key pro-inflammatory cytokine responsible for driving eosinophil differentiation and maturation from progenitor cells, but rather through eosinophil-derived interleukin-4 (IL-4) [13]. Given the evidence of potential eosinophilia-related cardiac complications, the eosinophil levels were monitored, and an increase in levels was observed. This is frequently observed during duplimab treatment.

Treatment options in patients with cardiovascular comorbidities pose a challenge for a clinician. Cyclosporine was not incorporated as a treatment option because of the long-term adverse effects associated with the drug, which could potentially exacerbate the patients’ comorbidities [14]. Instead, the treatment of choice for this patient was dupilumab in a dose of 600 mg followed by 300 mg every 2 weeks. The success of this treatment has been documented by previous studies with patients with AD and coexisting comorbidities. Dupilumab is a fully human monoclonal antibody that has been shown to reduce and downregulate pro-inflammatory cytokines such as IL-4 and IL-13, which are key mediators in AD and also involved in the pathogenesis of cardiovascular diseases and atherosclerosis [15,16]. This mechanism is not fully understood, and therefore, the improvements in cardiac function are not a causal relationship but more of an observed effect alongside the patient's other cardiologic medications. In terms of improvement of cardiac function, the use of heart failure medications like sacubitril/valsartan may have also contributed to improvements in cardiac function. However, existing studies suggest that its efficacy in improving EF is generally lower than what was observed in this case. For instance, Liu et al. reported an increase in left ventricular ejection fraction from approximately 35% to 50% over a six-month period, which is notably less than the improvement seen in our case [17].

The general safety profile of dupilumab is also considered, and although studies report frequent adverse effects, they are usually mild and can include eosinophilia, conjunctivitis, injection site reactions, and meibomianitis [15]. Dupilumab has also demonstrated a favorable safety profile in patients with various cardiovascular comorbidities, including those who have undergone heart transplantation. Studies by Sklover and Hamid have reported that patients with pre-existing cardiac conditions post-transplant not only experienced improvements in dermatological outcomes but also exhibited enhanced cardiac function, as evidenced by an elevation in EF [18,19].

In this case study, an initial dose of 600mg and the maintenance dose of 300 mg of dupilumab were also evaluated by a cardiologist so that it could be used without contraindications. The overall efficacy of the drug was represented by the considerable reduction in EASI scores from 67.8 to 3.2 and the DLQI, which decreased from 14 to 0. This was also seen in a placebo study by Thaçi et al., where the mean change in EASI and DLQI was 55.7% and 61.6%, respectively. Notably, the improvement in cardiac function is represented by the increase in ejection fraction from 21% to 61%, which signifies that dupilumab may have a beneficial impact on the overall health status [14]. The interdisciplinary integration between dermatology and cardiology was necessary in determining the contraindication to cyclosporine and the indication to initiate dupilumab. It additionally allows for the parameters of heart failure and AD to be monitored simultaneously. However, the collaboration also includes challenges whereby the cardiology records of ejection fraction across the entire period of time were not always accessible, which limits the longitudinal evaluation of cardiac function. Long-term cardiac health should also be routinely monitored using parameters such as left ejection fraction (LVEF) and biomarkers such as N-terminal pro-B-type natriuretic peptide (NT-proBNP). The most probable diagnosis, given the coexistence of allergic comorbidities, such as AD and asthma, is eosinophilic myocarditis, progressing to cardiomyopathy and heart failure. This diagnosis was based on clinical findings, and there was no biopsy to support this. Improvements in left ventricular EF and overall cardiac function could potentially be achieved through the introduction of novel heart failure therapies, including sacubitril/valsartan and dapagliflozin. However, the observed enhancement in cardiac function is likely attributable, at least in part, to the therapeutic effects of dupilumab. Therefore, clinicians often consider dupilumab as the first-choice treatment for complex cases of AD [14].

Although this case report presents evidence of clinical improvement in both cardiac and dermatological outcomes following dupilumab treatment, its generalisability to broader patient populations is inherently limited due to the nature of single-patient case studies. Such reports lack control groups, statistical power, and the ability to account for potential confounding variables. Comparison with existing literature further underscores the limited corroboration regarding cardiac outcomes. While substantial evidence supports the efficacy of dupilumab in improving dermatological indices (such as DLQI and EASI, as demonstrated in the systematic review and meta-analysis by Koskeridis et al., which reported a standardized mean difference in EASI of -0.98) there is insufficient evidence from randomized controlled trials to validate its use for cardiac indications [20].

Nonetheless, the findings of this case may hold particular clinical relevance for select patient subgroups, especially those with significant comorbidities who have failed conventional therapies or have contraindications to systemic immunosuppressants such as cyclosporine. While the upfront cost of dupilumab is relatively high, emerging evidence supports its overall cost-effectiveness, particularly when initiated early in the disease course. Early intervention may not only improve dermatological outcomes but also potentially reduce the long-term healthcare burden associated with comorbid conditions, including heart failure. This underscores the need for further investigation into the broader therapeutic benefits of dupilumab, including its possible role in improving cardiac outcomes and reducing associated treatment costs in complex patient populations.

## Conclusions

This case report presents how the problem of comorbidity in atopic dermatitis can be successfully managed using dupilumab therapy. For patients who don’t respond to conventional treatments or are not eligible for systemic cyclosporine, dupilumab is a successful treatment choice. It shows significant improvements in both dermatological and cardiovascular parameters. One aspect illustrated by this case report is the multidisciplinary approach required when choosing an effective management plan for AD patients with comorbidities.
